# A case of duodenal pyloric gland adenoma with high‐grade dysplasia arising from ectopic gastric mucosa

**DOI:** 10.1002/deo2.70135

**Published:** 2025-05-06

**Authors:** Kenichiro Nakachi, Hironobu Nagumo, Tetsuro Okura, Wataru Ohno, Takashi Ashikawa, Tomoyuki Funato, Toshiyasu Shiratori, So Nakaji, Shion Ando

**Affiliations:** ^1^ Gastroenterological Medicine Kameda Medical Center Chiba Japan; ^2^ Department of Pathology Kameda Medical Center Chiba Japan

**Keywords:** duodenal ectopic gastric mucosa, duodenal pyloric gland adenoma, endoscopic submucosal dissection, high‐grade dysplasia, narrow‐band imaging

## Abstract

Superficial non‐ampullary duodenal epithelial tumor is a rare disease, but its frequency has reportedly been increasing in recent years. We report a case of duodenal pyloric gland adenoma with high‐grade dysplasia arising from ectopic gastric mucosa. Esophagogastroduodenoscopy detected a 5‐mm raised lesion on the anterior surface of the duodenal bulb. The lesion was diagnosed as gastric foveolar metaplasia with biopsy. A second esophagogastroduodenoscopy was performed 13 years later. The nodule showed a two‐stage elevation and a biopsy revealed EGM. The lesion was followed up with EGD almost every year, with enlargement observed each time. Endoscopic submucosal dissection was performed. Histopathological examination revealed pyloric gland adenoma with high‐grade dysplasia. Ectopic gastric mucosa was observed in the tumor pathologically and transformation of the EGM into a tumor was followed endoscopically over time.

## INTRODUCTION

Superficial non‐ampullary duodenal epithelial tumor (SNADET) is a rare disease, but its frequency has reportedly been increasing in recent years. SNADETs are divided into intestinal and gastric types based on the mucous phenotype. Gastric phenotype‐dominant cases are less frequent but may have higher malignant potential.^1^ SNADETs with a gastric‐type phenotype derive from tumor‐like lesions such as Brunner's gland hyperplasia, ectopic gastric mucosa (EGM) and gastric metaplasia, foveolar‐hyperplastic polyp.^2^ Carcinogenesis with an EGM origin has also been reported, but the details are unclear.

We demonstrated successful treatment of duodenal adenocarcinoma arising from EGM after 20 years of observation.

## CASE REPORT

**FIGURE 1:**
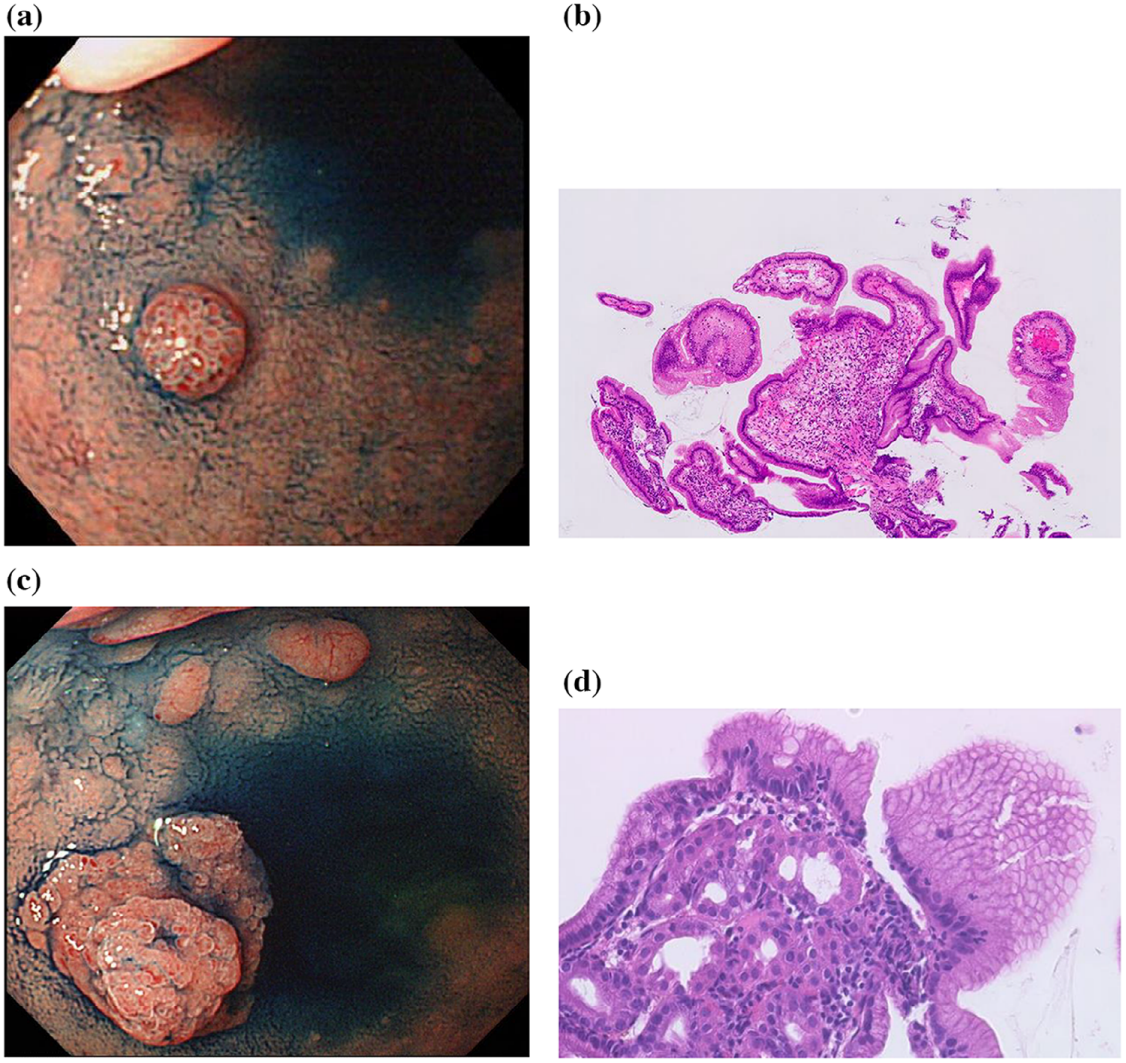
(a) Twenty years before the presentation of esophagogastroduodenoscopy. A 5 mm raised lesion on the anterior surface of the duodenal bulb. (b) Twenty years before the presentation of the histopathological image. A biopsy revealed gastric foveolar epithelial cells but no gastric fundic glands. (c) Seven years before the presentation of esophaogastoduodenoscopy displaying a double elevation. (d) Seven years before the presentation of the histopathological image. The image shows gastric fundic glands beneath the gastric foveolar epithelium.

A 75‐year‐old man with hypertension and hyperuricemia underwent esophagogastroduodenoscopy (EGD) that detected a 5‐mm raised lesion on the anterior surface of the duodenal bulb 20 years earlier, at 55 years old (Figure 1a). The reddish, raised lesion showed clear borders and was not accompanied by villous structures, unlike the surrounding duodenal mucosa. A biopsy revealed gastric foveolar epithelial cells but no gastric fundic glands (Figure 1b). At this point, the lesion was diagnosed as gastric foveolar metaplasia.

**FIGURE 2:**
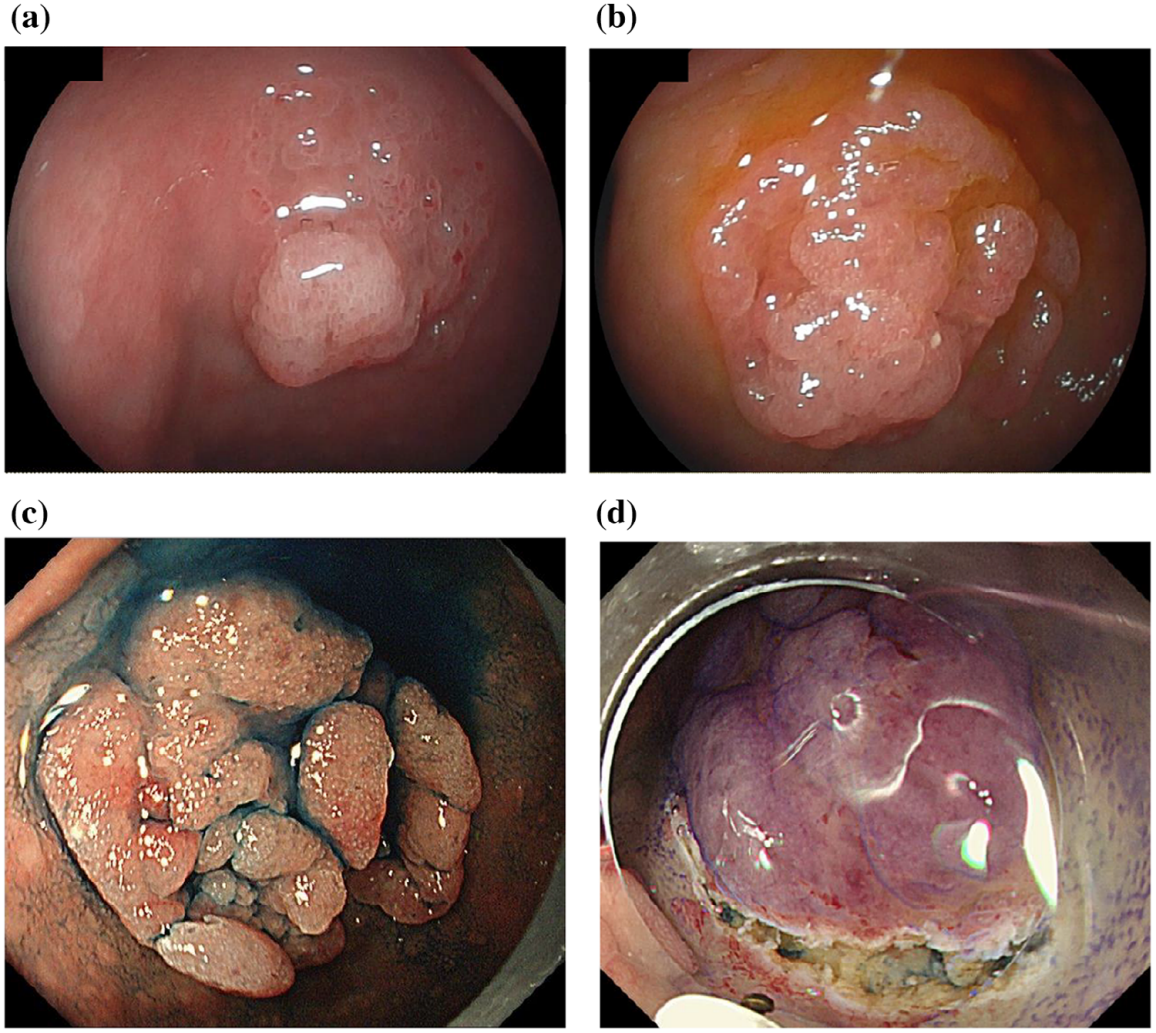
Progression of lesion. (a) Three years before the presentation. (b) One year before the presentation. (c) Immediately before endoscopic submucosal dissection. Chromoendoscopy with indigo carmine. (d) During endoscopic submucosal dissection after mucosal incision. 0.05% crystal violet is sprayed to detect the lesion before mucosal incision.

A second EGD was performed after about 13 years, at 67 years old. The nodule was enlarged and was surrounded by a flattened ridge, displaying a double elevation (Figure 1c). Biopsy revealed gastric foveolar epithelium and gastric fundic glands and EGM was diagnosed (Figure 1d). Follow‐up EGD was performed almost every year and enlargement was observed each time (Figure 2a,b).

**FIGURE 3:**
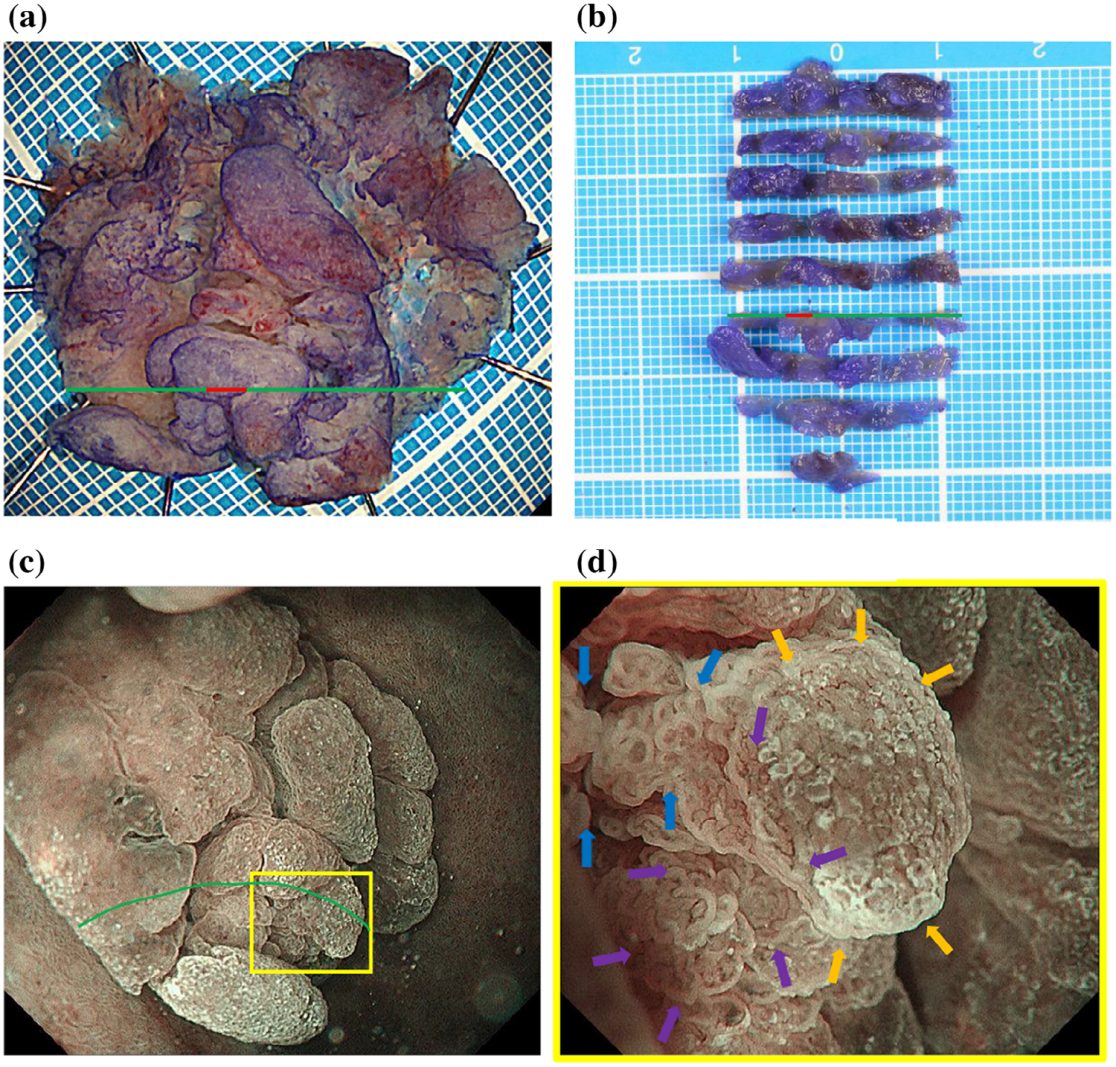
High‐grade dysplasia (red line) was observed in only one section (green line). (a) A macroscopic image of the resected specimen. (b) A macroscopic image of the sectioned specimen. (c) General view of the lesion using magnifying endoscopy with narrow‐band imaging of the lesion. (d) A magnified image of the lesion (box in part c) shows the vessels within the epithelial circle pattern suggesting a papillary structure (blue arrows). Furthermore, open‐loop‐type irregular vascular component within an irregular microsurface pattern composed of fused polygonal surface components is also observed (purple arrows). In addition, irregular, reticulated, and branched microvessels in the area with no apparent surface pattern are also observed (orange arrows).

The last EGD follow‐up detected that the lesion had grown to a maximum diameter of 25 mm (Figure 2c). Magnifying endoscopy with narrow‐band imaging (ME‐NBI) showed the vessels within an epithelial circle (VEC) pattern suggesting a papillary structure. Furthermore, an open‐loop‐type irregular vascular component within an irregular microsurface pattern composed of fused polygonal surface components was also observed. In addition, irregular, reticulated, and branched microvessels in the area with no apparent surface pattern were also observed (Figure 3d).

We eventually decided on endoscopic submucosal dissection (ESD) for diagnostic treatment.

ESD was performed using a DualKnife J (Olympus) with the GIF‐H290T electronic endoscope system (Olympus) and a monopolar cutting electrosurgical unit VIO 300D (Electromedizin). 0.05% crystal violet was sprayed to detect the lesion before mucosal incision. No severe bleeding occurred during ESD. En‐bloc resection of this lesion was accomplished without serious complications (Figure 3a,b).

**FIGURE 4:**
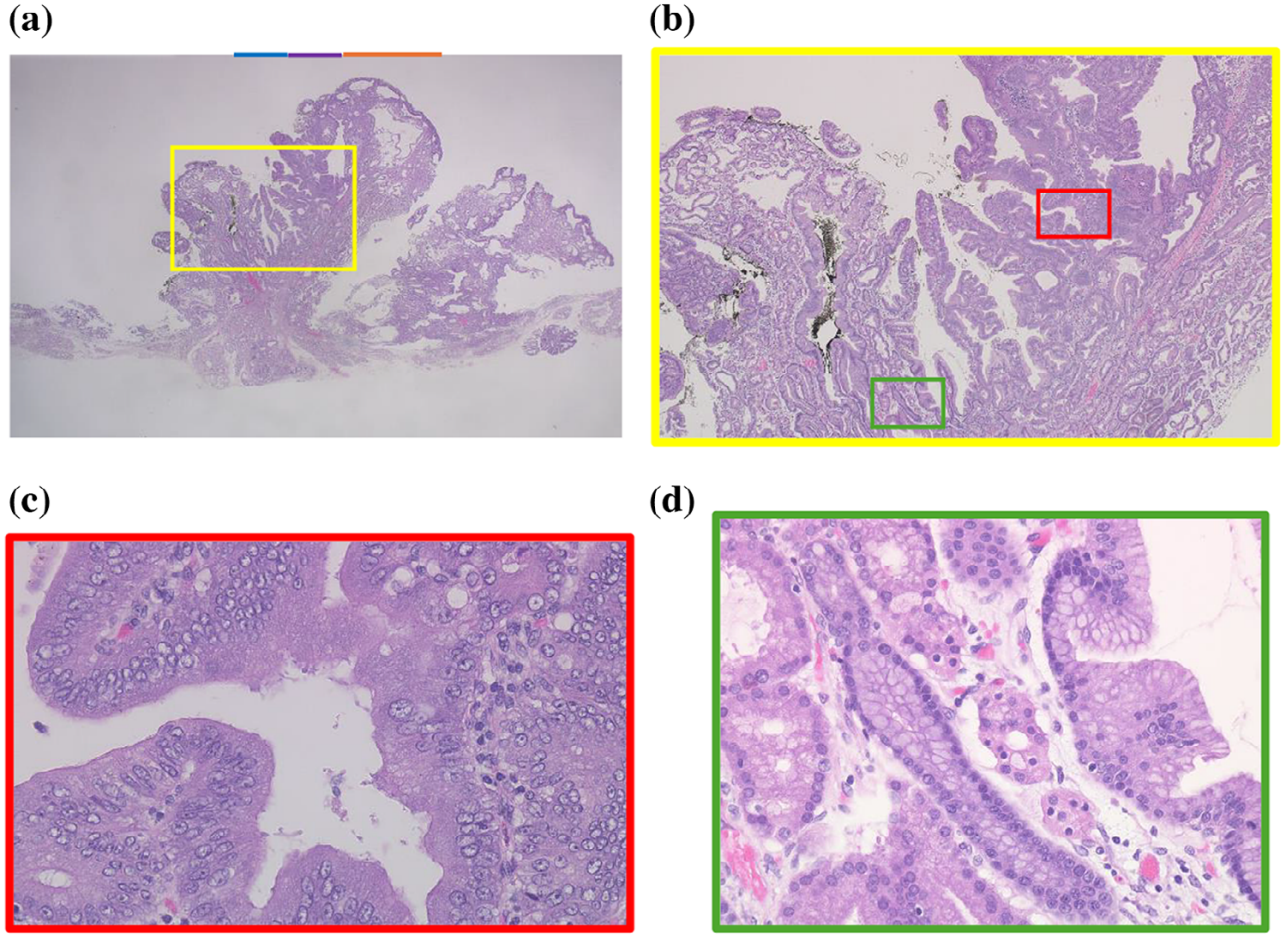
Histopathological image which is inverted left to right to match the orientation of the endoscopic image. (a) Low‐power image of the endoscopic submucosal dissection specimen (12.5×). (b) Higher magnification showing papillary proliferation of neoplastic pyloric‐type glands. (c) High‐grade dysplasia. Tumor cells show enlarged and euchromatic nuclei with prominent nucleoli and loss of polarity (400×). (d) Gastric fundic glands are observed within the tumor (400×).

Histopathological examination of the specimen from ESD revealed an elevated tumor comprising closely packed and focally dilated pyloric‐type glands, lined by cuboidal/low columnar epithelia (Figure 4a,b). The neoplastic cells exhibited a distinctive ground‐glass appearance, with cytoplasm that was clear to lightly eosinophilic. The tumor displayed a region of disrupted architecture, enlarged and euchromatic nuclei with prominent nucleoli, and loss of polarity (Figure 4c). Based on these findings, the diagnosis was pyloric gland adenoma (PGA) with high‐grade dysplasia (HGD). Immunohistochemistry revealed that the tumor expressed MUC6, while MUC5AC was predominantly expressed in the mucosal surface layer and in areas of HGD. MUC2 was negative (Figure S1). In addition, gastric fundic glands were observed within the tumor, indicating the transformation of the EGM into a tumor (Figure 4d). HGD was only observed in this section (Figure 4). The section was marked with a green line and the part with HGD with a red line in Figure 3a,b.

## DISCUSSION

The frequency of EGM is reportedly 0.5%–2%.^3^, ^4^ This pathology is typically recognized as single or multiple raised lesions with normal to erythematous coloration.

In the present case, the initial endoscopy showed a nodular ridge with a single erythematous tone, typical of EGM. However, the first biopsy revealed only foveolar epithelial cells and no gastric fundic glandular tissue, and gastric foveolar metaplasia was diagnosed. A second endoscopy showed a flattened elevation below the nodule, resulting in a two‐level elevation. Biopsy revealed gastric foveolar epithelium and gastric fundic glands, and EGM was histopathologically confirmed. The initial biopsy may have failed to collect fundic gland cells deep in the EGM. Hashimoto et al. reported that EGM might represent one type of a series of metaplastic changes that continue from gastric foveolar metaplasia.^5^ Which of these options apply in the present case is unclear.

In the present case, the tumor was thought to have originated from the deep gastric fundic gland beneath a nodule of foveolar epithelium and enlarged like a flattened ridge. Gastric PGA has been clarified to represent a tumor associated with the proliferation and differentiation of the mucosal neck cell/chief cell lineage constituting the fundic gland.^6^ The present case demonstrated that the same thing occurs in the EGM of the duodenum from the deep gastric fundic glands. The change from EGM to pyloric dysplasia has been followed for 20 years, and changes were therefore able to be observed in detail.

Using ME‐NBI, gastric PGA showed an irregular microvascular architecture composed of closed‐ or open‐loop‐type vascular components, plus an irregular microsurface pattern composed of fused polygonal surface components and a conspicuous VEC pattern.^7^ The same findings were seen in the vessels within an open‐loop‐type irregular vascular component within an irregular microsurface pattern composed of a fused polygonal surface component and VEC pattern in the duodenum in the present case (Figure 3d).

On the other hand, there was a wide area lacking the microsurface pattern with long, branched microvessels in the present case (Figure 3d), although there are no reports of the same finding in gastric PGA. This ME‐NBI finding might be specific to PGA in the duodenum. The irregular, reticulated, and branched microvessels in the area surrounded by orange arrows in Figure 3d with no apparent surface pattern coincide with the orange line in Figure 4a. The pathology specimen showed pyloric gland ducts running laterally and not open. Therefore, a microsurface pattern was not observed in the endoscopic examination.

The specimen also showed HGD. The area of blue arrows in Figure 3d coincided with the blue line in Figure 4a and the area of purple arrows coincided with the purple line. HGD was present in the areas of blue and purple arrows. Therefore, the VEC pattern or open‐loop‐type irregular vascular component within an irregular microsurface pattern composed of fused polygonal surface component was thought to represent HGD.

Although cases of duodenal cancer arising from EGM have been reported, all were diagnosed based on pathological findings showing the EGM and duodenal cancer adjacent to each other.^8^, ^9^, ^10^ In this case, EGM was observed in the tumor pathologically and transformation of the EGM into a tumor was able to be followed endoscopically over time.

Although the relationship between EGM and the development of adenomas and adenocarcinoma is still unclear, Sawada et al. reported gastric‐type duodenal neoplasms with rapid growth suspected to arise from EGM.^2^ Regular endoscopic follow‐up is necessary for EGM and biopsy or diagnostic treatment should be considered if changes are detected. Essentially, further cases need to be accumulated to clarify the relationship between EGM and the development of adenomas and adenocarcinomas.

## CONFLICT OF INTEREST STATEMENT

None.

## Supporting information




**FIGURE S1** The tumor expresses MUC6, while MUC5AC is predominantly expressed in the mucosal surface layer and in areas of high‐grade dysplasia (HGD). MUC2 is negative (40×).
