# Supplementary figures and images for: A case of duodenal pyloric gland adenoma with high‐grade dysplasia arising from ectopic gastric mucosa
Source: DEN Open. 2025 May 6;6(1):e70135. doi: 10.1002/deo2.70135 (PMC12054478; doi:10.1002/deo2.70135)

## Slide 1
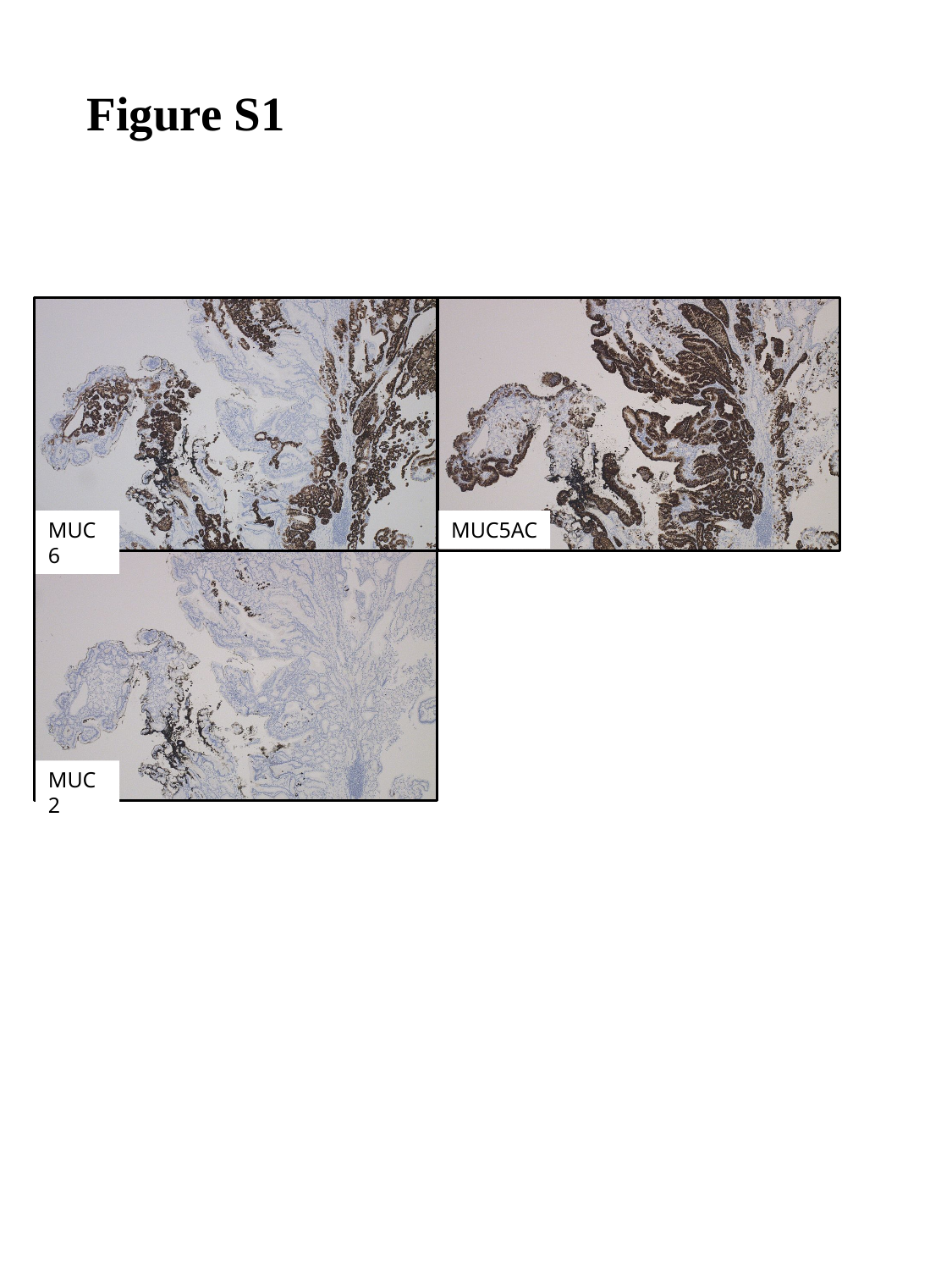

Figure S1
MUC5AC
MUC6
MUC2

Supplement: Supplementary file 1 — FIGURE S1 The tumor expresses MUC6, while MUC5AC is predominantly expressed in the mucosal surface layer and in areas of high‐grade dysplasia (HGD). MUC2 is negative (40×). [file DEO2-6-e70135-s001.pptx]
